# Identification and Expression Analysis of the Genes Involved in the Raffinose Family Oligosaccharides Pathway of *Phaseolus vulgaris* and *Glycine max*

**DOI:** 10.3390/plants10071465

**Published:** 2021-07-16

**Authors:** Ramon de Koning, Raphaël Kiekens, Mary Esther Muyoka Toili, Geert Angenon

**Affiliations:** 1 Research Group Plant Genetics, Vrije Universiteit Brussel, 1050 Brussels, Belgium; Ramon.De.Koning@vub.be (R.d.K.); Raphael.Kiekens@vub.be (R.K.); Mary.Esther.Muyoka.Toili@vub.be (M.E.M.T.); 2 Department of Horticulture, Jomo Kenyatta University of Agriculture and Technology, Juja 01001, Kiambu County, Kenya

**Keywords:** common bean, Fabaceae, legumes, qRT-PCR, raffinose family oligosaccharides (RFOs), RNA-seq, soybean

## Abstract

Raffinose family oligosaccharides (RFO) play an important role in plants but are also considered to be antinutritional factors. A profound understanding of the galactinol and RFO biosynthetic gene families and the expression patterns of the individual genes is a prerequisite for the sustainable reduction of the RFO content in the seeds, without compromising normal plant development and functioning. In this paper, an overview of the annotation and genetic structure of all galactinol- and RFO biosynthesis genes is given for soybean and common bean. In common bean, three galactinol synthase genes, two raffinose synthase genes and one stachyose synthase gene were identified for the first time. To discover the expression patterns of these genes in different tissues, two expression atlases have been created through re-analysis of publicly available RNA-seq data. De novo expression analysis through an RNA-seq study during seed development of three varieties of common bean gave more insight into the expression patterns of these genes during the seed development. The results of the expression analysis suggest that different classes of galactinol- and RFO synthase genes have tissue-specific expression patterns in soybean and common bean. With the obtained knowledge, important galactinol- and RFO synthase genes that specifically play a key role in the accumulation of RFOs in the seeds are identified. These candidate genes may play a pivotal role in reducing the RFO content in the seeds of important legumes which could improve the nutritional quality of these beans and would solve the discomforts associated with their consumption.

## 1. Introduction

The common bean (*Phaseolus vulgaris*) and soybean (*Glycine max*) are of outstanding importance for human and animal nutrition. They have a high nutritional quality, containing large amounts of proteins, carbohydrates, vitamins, minerals and dietary fibers. Beans are used as a major source of calories and proteins in developing countries, and they are the main legumes in vegetarian diets [1,2,3,4]. Furthermore, diets consisting mainly of cereals can be complemented with legumes to increase the essential nutrients acquired, making them ideal components to include in our diets [5]. 

A major drawback of the consumption of beans in large quantities is the presence of several anti-nutritional factors. Beans contain high amounts of raffinose family oligosaccharides (RFOs) [1,6,7,8,9]. These sugars contain one or more α-1,6 galactosyl bonds which make them indigestible for humans and monogastric animals because they lack α-galactosidase, the enzyme required to break down these sugars. Instead, these sugars undergo fermentation by gut microbiota in the large intestine resulting in the production of gases such as methane and carbon dioxide [1,10]. The production of gases does not pose any problems for humans or animals when small quantities of beans are consumed. On the contrary, the breakdown of small amounts of RFOs might stimulate the growth of beneficial bacteria in the large intestine providing RFOs a potential small probiotic effect [11,12]. However, the consumption of large amounts of beans results in the production of gases which leads to unwanted flatulence and digestive disturbances [13]. The presence of RFOs in the feed also results in shorter transit times through the digestive tract, reducing the absorption of nutrients and causing poor weight gain [14,15]. Lowering the amount of RFOs in the seeds could improve the nutritional quality of these beans and would solve the discomforts associated with their consumption [14]. Since RFOs have many functions in the plant, a thorough understanding of the galactinol and RFO biosynthetic gene families and the expression patterns of the individual genes is needed to be able to reduce the RFO content in the seeds, without compromising normal plant development and functioning. 

RFOs play an important role in the transport and storage of carbon within the plant [16]. Sucrose produced as a result of photosynthesis in mesophyll cells (source), can move via the bundle sheath cells located at the minor veins, into specialized companion cells which are also known as intermediary cells. Here RFO biosynthetic enzymes are present which convert sucrose into RFOs. The RFOs are then loaded into the phloem according to a polymer trapping model [17]. This model specifies that, due to the large size of the RFOs, the diffusion back into the mesophyll cells is prevented. The osmotic pressure facilitates the migration towards the sieve elements where the RFOs are then transported to other parts of the plant (sinks) where they can be broken down by alkaline α-galactosidase [16,17,18,19,20]. The production of RFOs in the intermediary cells allows plants to maintain a high sugar concentration in the phloem [19]. It is also suggested that RFOs play a role in the abiotic stress responses of plants. This is mainly based on the observations that the RFO content increases in plants as a response to various stresses such as drought, heat, cold and salt stress [21,22,23,24,25,26]. Several different mechanisms in which RFOs provide protection against abiotic stress have been proposed. During abiotic stress, RFOs could stabilize membranes and proteins with hydrophilic interactions, act as reactive oxygen species scavengers or serve as osmolytes to reduce water loss [25,27,28,29,30]. In leguminous crops, RFOs are mainly stored in the seeds under normal growth conditions. They are produced de novo during the maturation of the seed and protect the seed against seed desiccation and provide seed longevity for storage [31,32,33,34,35,36]. During germination, they are rapidly broken down by acidic α-galactosidases providing energy and carbon to the young seedling [37,38]. However, it needs to be noted that they are not essential for germination in soybean [39].

Four different members of the RFO family are found in plants: raffinose, stachyose, verbascose and ajugose, which are respectively, tri-, tetra-, penta- and hexasaccharides [37]. Soybean and common bean primarily produce, raffinose and stachyose, and to a lesser extent, verbascose in their seeds [10,14]. The production of RFOs is mainly dependent on the influx of galactinol which functions as a galactosyl donor for the RFO synthesis. Galactinol is produced by galactinol synthase (GolS) which catalyzes the galactosyl transfer from uridine diphosphate galactose to myo-inositol forming galactinol [37]. Raffinose synthase (RS) catalyzes the formation of raffinose, by transferring a galactosyl moiety from galactinol to sucrose. This reversible reaction produces the first RFO in the RFO biosynthesis pathway [40]. The larger RFOs, stachyose and verbascose, can both be formed by stachyose synthase (SS) which has, contrary to RS, a broader substrate specificity [41]. For the formation of stachyose and subsequently verbascose, SS uses respectively, raffinose or stachyose as a galactosyl acceptor. As a galactosyl donor, either galactinol, stachyose or galactosyl cyclitols can be used. Furthermore, SS also has the ability to synthesize these galactosyl cyclitols by facilitating the galactosyl transfer from galactinol to a cyclitol [37]. Galactosyl cyclitols contain, like RFOs, an α-1,6 bond. It is important to point out that galactosyl cyclitols potentially have a similar function as RFOs during seed development due to their high similarity in structure and contribute to the digestive problems humans and monogastric animals face after consumption of beans [37,42]. An alternative route to produce higher RFOs (stachyose, verbascose and ajugose), independent of galactinol, has only been reported in two species of the Lamiaceae family (*Ajuga reptans* and *Coleus blumei*) where the enzyme galactan:galactan galactosyltransferase (GGT) catalyzes the transfer of a galactosyl moiety from one RFO to another [43,44]. The GGT enzyme has only been found in the vacuoles of leaves, which makes the synthesis of higher RFOs in seeds unlikely to be dependent on this pathway [41]. 

Previous attempts to lower the amount of RFOs in the seeds have shown to be successful in *G. max*. Valentine et al. (2017) reduced the raffinose and stachyose content in seeds from respectively, 0.63% and 3.79% in the wild type to 0.11% and 1.21% in a transgenic line using a silencing construct targeting a RS isoform [14]. In a recent study by Le et al. (2020) two GolS genes were knocked out, reducing the total RFO content by 35.2% [45]. However, more progress can be made to lower the RFO content in the seeds more efficiently without compromising normal plant development and functioning. An in-depth study of all genes involved can elucidate which strategies can be best perused to achieve this. In contrast to *G. max*, the RFO biosynthesis pathway is still understudied in *P. vulgaris*. In this paper, an overview of the annotation and genetic structure of all galactinol- and RFO biosynthesis genes is given for soybean and common bean. In *P. vulgaris*, three GolS genes, two RS genes and one SS gene were identified for the first time. To discover the expression patterns of these genes in different tissues, two expression atlases have been created through a re-analysis of publicly available RNA-seq studies. Furthermore, little is known about the expression patterns of these galactinol- and RFO biosynthesis genes during seed development in common bean. De novo expression analysis through an RNA-seq study during seed development in three varieties of common bean gave more insight into the expression patterns of these genes and seed specific genes involved in the RFO production have been identified. The results of the expression analysis indicate tissue-specific expression patterns of different classes of galactinol- and RFO synthase genes in soybean and common bean. With the obtained knowledge, suitable candidate genes to alter the expression levels of the galactinol- and RFO synthase genes are proposed to lower the amount of RFOs in the seeds. 

## 2. Results

### 2.1. In Silico Identification of the Galactinol- and RFO Biosynthetic Enzymes in Phaseolus vulgaris and Glycine max

**Figure 1:**
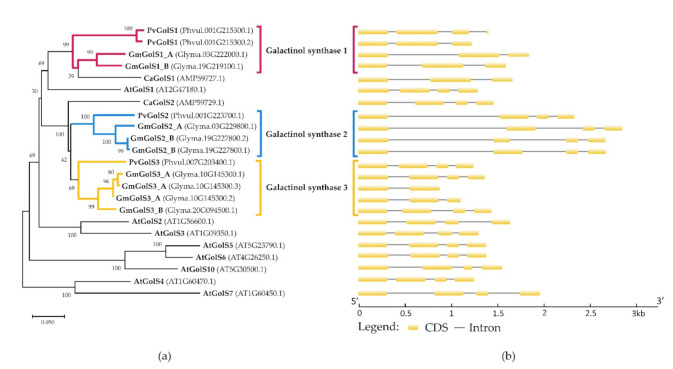
Analysis of the phylogenetic relationships and gene structures of galactinol synthase (GolS). (**a**) Evolutionary relationship of GolS enzymes of *G. max*, *P. vulgaris* and other well-characterized taxa (*A. thaliana* and *C. arietinum)*. The GolS enzymes of *P. vulgaris* and *G. max* are subdivided into three different classes based on their clustering (GolS1, GolS2 and GolS3. The phylogenetic tree was made in MEGA X (v10.2.4) using the neighbor-joining algorithm combined with a bootstrap test of 1000 replicates (next to the branches, the percentage of replicate trees in which the associated taxa clustered together is shown) [46,47,48]. The Poisson correction method was used to compute the evolutionary distances with the number of amino acid substitutions per site as a unit [49]. The phylogenetic tree was drawn to scale using the calculated evolutionary distances as branch lengths. This analysis involved 23 amino acid sequences. For each sequence pair, all ambiguous positions were removed using the pairwise deletion option and the final dataset consisted of 355 positions. The multiple sequence alignment was created using the MUSCLE algorithm; (**b**) structures of genes encoding the GolS enzymes were visualized using GSDS software [50].

Using a customized bioinformatics pipeline, three GolS genes were identified in *P. vulgaris*, (Phvul.001G215300, Phvul.001G223700 and Phvul.007G203400) and six GolS genes in *G. max* (Glyma.03G229800, Glyma.19G227800, Glyma.20G094500, Glyma.03G222000, Glyma.19G219100 and Glyma.10G145300). Some genes encode multiple predicted isoforms. In *P. vulgaris*, only for Phvul.001G215300 two isoforms are predicted. In *G. max*, two isoforms for Glyma.19G227800 and three isoforms for Glyma.10G145300 are predicted. A phylogenetic tree has been made using the amino acid sequences of the predicted proteins of the GolS enzymes of *P. vulgaris*, *G. max* and well-characterized GolS enzymes of Arabidopsis thaliana and Cicer arietinum (Figure 1a). Furthermore, the gene structures of the GolS enzymes are presented in Figure 1b. Based on the clustering of the *P. vulgaris* and *G. max* enzymes in the phylogenetic tree, the GolS enzymes of *G. max* and *P. vulgaris* were categorized into three classes: galactinol synthase 1 (GolS1), galactinol synthase 2 (GolS2) and galactinol synthase 3 (GolS3). The predicted primary transcript isoforms of the GolS1 genes consist of three exons and two introns. The GolS2 and GolS3 genes consist of four exons and three introns. 

**Figure 2:**
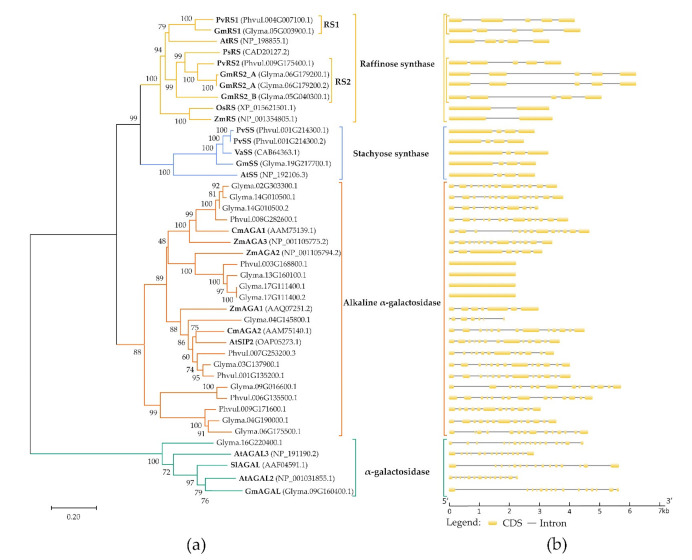
Analysis of the phylogenetic relationships and gene structures of the raffinose family oligosaccharides’ biosynthetic and catabolic enzymes. (**a**) Evolutionary relationship of the raffinose synthase (RS), stachyose synthase (SS) and (alkaline) α-galactosidase (AGA) enzymes of *G. max*, *P. vulgaris* and other well-characterized enzymes of different taxa (*A. thaliana*, *P. sativum*, *V. angularis*, *S. lycopersicum*, *O. sativa* and *Z. mays)*. The RS enzymes of *P. vulgaris* and *G. max* are subdivided into two different classes (RS1 and RS2) based on their clustering. The phylogenetic tree was made in MEGA X (v10.2.4) using the neighbor-joining algorithm combined with a bootstrap test of 1000 replicates (next to the branches are the percentage of replicate trees in which the associated taxa clustered together is shown) [46,47,48]. The Poisson correction method was used to compute the evolutionary distances with the number of amino acid substitutions per site as a unit [49]. The phylogenetic tree was drawn to scale using the calculated evolutionary distances as branch lengths. This analysis involved 43 amino acid sequences. For each sequence pair, all ambiguous positions were removed using the pairwise deletion option and the final dataset consisted of 1030 positions. The multiple sequence alignment was created using the MUSCLE algorithm. (**b**) Structures of genes encoding the RFO synthase enzymes were visualized using GSDS software [50].

**Figure A1:**
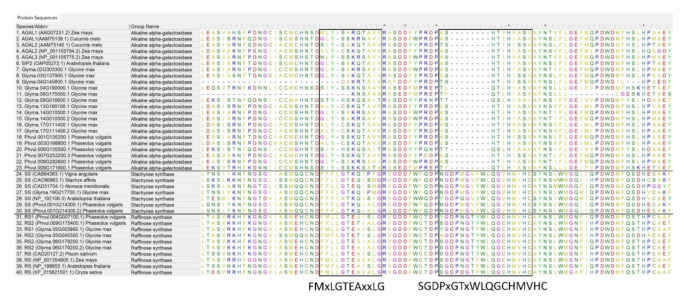
Visualization of the amino acid motifs present in raffinose synthase (RS) but absent in alkaline α-galactosidase. A multiple sequence alignment was performed using the MUSCLE algorithm in MEGA X of the protein sequences of potential RFO synthase enzymes of *G. max* and *P. vulgaris* together with well-characterized RFO synthase and alkaline α-galactosidase enzymes of *O. sativa*, *C. melo*, *Z. mays*, *A. thaliana*, *P. sativum*, *V. angularis*, *Stachys affinis* and *Alonsoa meridionalis*. To distinguish RS from alkaline α-galactosidase, two conserved motifs, FMxLGTEAxxLG and SGDPxGTxWLQGCHMVHC, were used. These motifs are present in the amino acid sequence of RS but absent in alkaline α-galactosidase [51].

**Figure A2:**
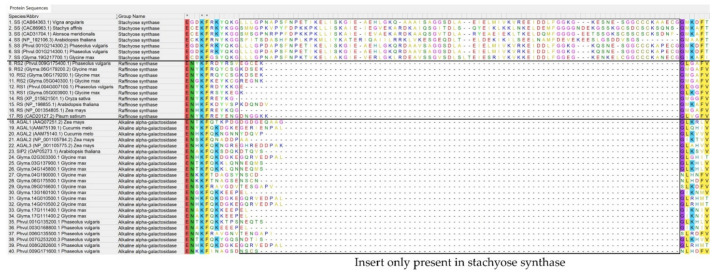
Visualization of the amino acid insert only present in stachyose synthase (SS). A multiple sequence alignment was performed using the MUSCLE algorithm in MEGA X of the protein sequences of potential RFO synthase enzymes of *G. max* and *P. vulgaris* together with well-characterized RFO synthase and alkaline α-galactosidase enzymes of *O. sativa*, *C. melo*, *Z. mays*, *A. thaliana*, *P. sativum*, *V. angularis*, *S. affinis* and *A. meridionalis*. RS can be distinguished from SS because of an insert present only in SS [52].

Nine possible RFO biosynthesis genes have been predicted in *P. vulgaris* and fourteen in *G. max* using our bioinformatics pipeline. Most proteins encoded by these genes are annotated as potential RFO biosynthesis enzymes but could also be wrongly annotated. To distinguish RS from alkaline α-galactosidase two conserved motifs (FMxLGTEAxxLG and SGDPxGTxWLQGCHMVHC) were used (Figure A1 in Appendix A). These motifs are present in the amino acid sequence of RS but absent in alkaline α-galactosidase [51]. RS can be distinguished from SS because of an amino acid insert present only in SS (Figure A2 in Appendix A) [40,52]. A phylogenetic tree of the amino acid sequences has been made to evaluate the evolutionary relationship of the different RFO biosynthetic and hydrolytic enzymes of *P. vulgaris*, *G. max* and other well-characterized taxa: *A. thaliana*, *Pisum sativum*, *Vigna angularis*, *Solanum lycopersicum*, *Oryza sativa* and *Zea mays* (Figure 2a). The phylogenetic tree clusters 4 different groups of enzymes with high certainty (bootstrap score > 88): RS, SS, alkaline α-galactosidase and α-galactosidase. In addition, gene features of these enzymes were visualized (Figure 2b). The RS genes in *G. max* and *P. vulgaris* all consist of 5 exons and 4 introns. The genes encoding SS consist of 4 exons and 3 introns. This is in contrast with the alkaline α-galactosidase genes which mostly consist of 13 exons although quite a large variation can be seen in this group. Some of the alkaline α-galactosidase genes consist of 1, 7, 12 or 14 predicted exons. From these results, it can be concluded that *P. vulgaris* most likely contains at least two RS genes (Phvul.009G175400 and Phvul.004G007100) and only one SS gene (Phvul.001G214300). *G. max* has three RS genes (Glyma.05G003900, Glyma.06G179200 and Glyma.05G040300) and one SS gene (Glyma.19G217700). Based on the phylogenetic tree, the RS enzymes of *G. max* and *P. vulgaris* have consequently been categorized into two classes: raffinose synthase 1 (RS1) and raffinose synthase 2 (RS2). An overview of the genes involved in the RFO biosynthesis pathway in *G. max* and *P. vulgaris* can be found in Table 1. The corresponding names of the genes found in Table 1 will be used in the rest of the text.

### 2.2. Transcriptomic Analysis of the RFO Biosynthetic Pathway

#### 2.2.1. Expression Analysis of Galactinol- and RFO Biosynthesis Genes in *Glycine max* and *Phaseolus vulgaris* by RNA-seq Re-Analysis

**Figure 3:**
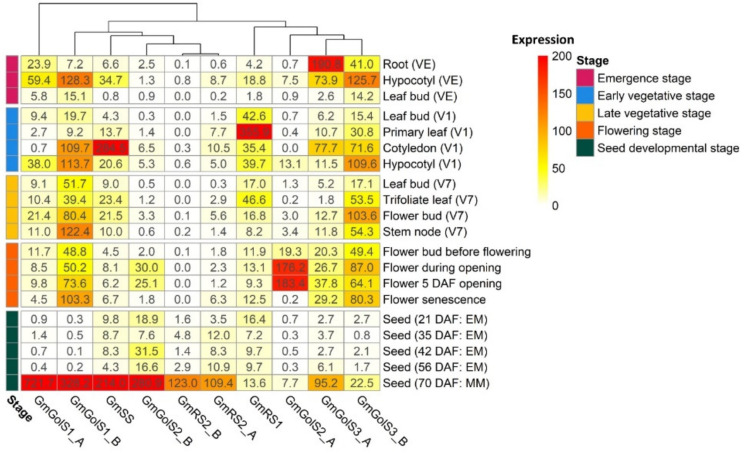
Heatmap of the normalized expression values (TPM) of the galactinol synthase (*GmGolS1-3*), raffinose synthase (*GmRS1-2*) and stachyose synthase (*GmSS*) genes in various plant tissues during different developmental stages in *G. max* cv. Wm82 (SRP038111). The rows represent plant tissues in five different developmental stages: emergence stage, early vegetative stage, late vegetative stage, flowering stage and seed developmental stage. More detailed information of the exact growth stage of the tissues can be found after the tissues names between brackets, with growth stage abbreviations (VE: emergence stage; V1: first-node stage; V7: seventh-node stage; EM: early maturation stage of the seed; MM: mid maturation stage of the seed; DAF: days after flowering) adapted from Fehr et al. [55]. The columns represent the different genes and their expression levels, represented with a color gradient in which white indicates no expression (0 TPM) and red the highest expression (200+ TPM). Hierarchical clustering was performed between the columns to obtain gene clusters.

**Figure 4:**
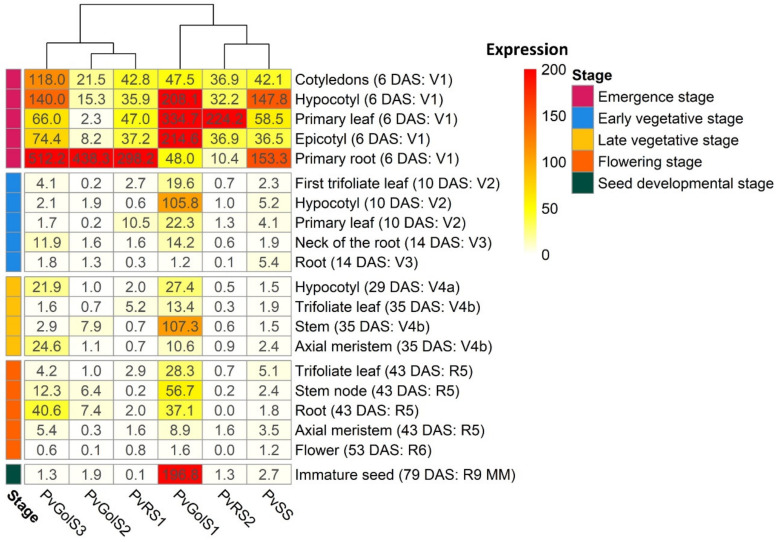
Heatmap of the normalized expression values, presented in transcripts per million (TPM), of the galactinol synthase (*PvGolS1-3*), raffinose synthase (*PvRS1-2*) and stachyose synthase (*PvSS*) genes in various plant tissues during different developmental stages in *P. vulgaris* cv. BAT93 (SRP030614). The rows represent plant tissues in five different developmental stages: emergence stage, early vegetative stage, late vegetative stage, flowering stage and seed developmental stage. More detailed information of the exact growth stage of the tissues can be found after the tissues name between brackets, indicated in days after sowing (DAS) and with growth stage abbreviations (V1: emergence stage; V2: primary leaves stage; V3: first trifoliate leaf stage; V4: third trifoliate leaf stage; R5: preflowering stage; R6: flowering stage; R9: maturity stage; MM: mid maturation stage of the seed) adapted from Vlasova et al. [53]. The columns represent the different genes and their expression levels, represented with a color gradient in which white indicated no expression (0 TPM) and red the highest expression (200+ TPM). Hierarchical clustering was performed between the columns to obtain gene clusters.

An expression atlas has been made from the RNA-seq data of studies SRP038111 (*G. max* cv. Wm82) and SRP030614 (*P. vulgaris* cv. BAT93) [53,54]. The normalized expression data, represented as transcripts per million (TPM), of the different GolS, RS and SS genes is shown in Figure 3 for *G. max* cv. Wm82 and Figure 4 for *P. vulgaris* cv. BAT93 during five different growth stages. The different classes of GolS (GolS1, GolS2 and GolS3) and RS (RS1 and RS2), as defined by the phylogenetic tree analysis, show tissue-specific expression patterns for both *G. max* cv. Wm82 and *P. vulgaris* cv. BAT93.

The normalized expression data of RNA-seq study SRP038111 in *G. max* cv. Wm82 shows that the GolS1 genes (GmGolS1_A and GmGolS1_B) are expressed highly in the hypocotyl during the emergence stage (resp. 59.4 and 128.2 TPM) and early vegetative stage (resp. 38.0 and 113.7 TPM). During the late vegetative stage, both genes are expressed in different types of tissues; GmGolS1_B, however, is mainly expressed in the stem node (122.4 TPM), flower bud (80.5 TPM), leaf bud (51.6 TPM) and trifoliate leaf (39.4 TPM) during the late vegetative stage. Furthermore, GmGolS1_B shows a higher expression in all stages of flower development. These two genes are highly expressed during the mid-maturation stage of the seed, with GmGolS1_A being expressed two times higher than GmGolS1_B in the seeds with expression levels of 721.7 TPM and 328.2 TPM, respectively. At the mid-maturation stage of the seed, both genes are expressed more in comparison with any other galactinol or RFO synthase gene. When observing the GolS2 genes (GmGolS2_A and GmGolS2_B) it can be seen that GmGolS2_A is highly expressed in the flower during the flowering stage, with expression levels of 176.2 TPM during the opening and 183.4 TPM five days after the opening of the flower. During the other developmental stages, no significant expression is observed. GmGolS2_B is mainly expressed in the seeds during the mid-maturation stage of the seeds (280.9 TPM) and less pronounced in the flower during the opening (30.0 TPM) and five days after the opening (25.1 TPM). The GolS3 genes (GmGolS3_A and GmGolS3_B) are both expressed in the roots (resp. 190.8 and 41.0 TPM) and hypocotyl (resp. 73.9 and 125.7 TPM) during the emergence stage. GmGolS3_B is mainly expressed in the hypocotyl (109.6 TPM) and to a lower extent in the primary leaf (30.8 TPM) during the early vegetative stage. In the late vegetative stage, GmGolS3_B is mainly expressed in the flower bud (103.6 TPM), stem node (54.3 TPM) and trifoliate leaf (53.5 TPM). During the flowering stage, both genes are expressed in the flower bud (resp. 20.3 and 49.4 TPM) and in the flower during the opening (resp. 26.7 and 87.0 TPM), 5 days after the opening (resp. 37.8 and 64.1 TPM) and during the senescence (resp. 29.2 and 80.3 TPM). During the mid-maturation stage of the seed, GmGolS3_A is primarily expressed (95.2 TPM). The soybean RS1 gene, GmRS1, is highly expressed in the primary leaf (355.9 TPM) during the early vegetative stage and in the trifoliate leaf (46.6 TPM) in the late vegetative stage. In the early vegetative stage, it is also expressed in the leaf bud (42.6 TPM) and hypocotyl (39.7 TPM). During the seed developmental stage, it is almost not expressed in the seed (13.6 TPM). This is in contrast with the RS2 genes (GmRS2_A and GmRS2_B) that are only expressed in the seed during the mid-maturation stage of the seed (resp. 123.0 and 109.4 TPM). In soybean, the SS gene, GmSS, is expressed in the hypocotyl during the emergence stage and early vegetative stage (resp. 34.8 and 20.6 TPM). During the late vegetative stage, it is expressed in the trifoliate leaf (23.4 TPM) and flower bud (21.5 TPM). In the seed developmental stage, it is expressed in the seed during the mid-maturation stage of the seed (214.0 TPM). The hierarchical clustering of the genes in the heatmap in Figure 3 also shows that the different GolS, RS and SS genes within one class have tissue-specific expression patterns and generally cluster close together. However, the GS2 genes do not cluster together mainly because of the expression difference in the seeds. 

In *P. vulgaris* cv. BAT93, the normalized expression data of the RNA-seq study SRP030614 (Figure 4) shows that PvGolS1 is expressed mainly in the primary leaf (334.7 TPM), epicotyl (214.6 TPM), hypocotyl (208.1 TPM) and to a lesser extent in the primary root (48.0 TPM) and cotyledons (47.5 TPM) during the emergence stage. During the early vegetative stage, it is mainly expressed in the hypocotyl (105.8 TPM) and to a lesser extent in the primary leaf (22.3 TPM), first trifoliate leaf (19.7 TPM) and the neck of the root (14.2 TPM). During the late vegetative stage, it is mainly expressed in the stem (107.3 TPM). During this stage, expression can also be seen in the hypocotyl (27.4 TPM) and trifoliate leaf (13.4 TPM) and the axial meristem (10.6 TPM). In the flowering stage, it is expressed in the stem node (56.7 TPM), the root (37.1 TPM), the trifoliate leaf (28.3 TPM) and axial meristem (8.9 TPM). During the seed developmental stage, it is the only gene of this pathway that is expressed in the seed during the mid-maturation stage (196.8 TPM). In addition, PvGolS1 shows the most TPM variation throughout the tissues in comparison with the other genes analyzed. Furthermore, in the primary root, a contrasting expression is seen, with low PvGolS1 expression compared to higher expression for all other genes except PvRS2. The common bean GolS2 gene, PvGOLS2, is almost exclusively expressed in the primary root (438.26 TPM) during the emergence stage. The GolS3 gene, PvGolS3, is also primarily expressed in the primary root (512.2 TPM) and to a lesser extent in the hypocotyl (140.0 TPM), the cotyledons (118.0 TPM), epicotyl (74.4 TPM) and primary leaf (66.0 TPM) during the emergence stage. In the early vegetative stage, it is expressed in the neck of the root (11.9 TPM) and during the late vegetative stage, expression is observed in the axial meristem (24.6 TPM) and hypocotyl (21.9 TPM). During the flowering stage, PvGolS3 is also mainly expressed in the roots (40.6 TPM) and to a lesser extent in the stem node (12.3 TPM). In common bean, the RS1 gene, PvRS1, is primarily expressed in the primary root (298.2 TPM) during the emergence stage and to a lesser extent in the primary leaf (47.0 TPM), the cotyledons (42.8 TPM), the epicotyl (37.2 TPM) and hypocotyl (35.9 TPM). During the early vegetative stage, it is expressed in the primary leaf (10.5 TPM) and during the late vegetative stage in the trifoliate leaf (5.3 TPM). PvRS2 is only expressed during the emergence stage, where it shows expression in the primary leaf (224.2 TPM) and to a lesser extent in the epicotyl (36.9 TPM), cotyledons (36.9 TPM) and hypocotyl (32.2 TPM). Finally, the common bean SS gene, PvSS, is primarily expressed in the primary root (153.3 TPM) and hypocotyl (147.8 TPM) and to a lesser extent in the primary leaf (58.5 TPM), cotyledons (42.1 TPM) and epicotyl (36.5 TPM) during the emergence stage.

#### 2.2.2. *De Novo* Expression Analysis of Galactinol- and RFO Biosynthesis Genes during Seed Development in *P. vulgaris*

**Figure 5:**
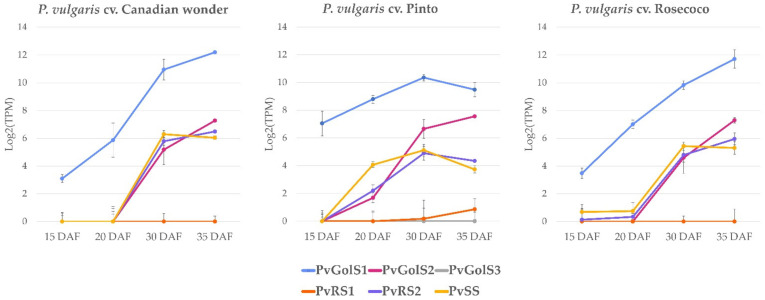
RNA-seq expression analysis of the galactinol- and RFO biosynthesis genes during seed development in *P. vulgaris* cvs. Canadian wonder, Pinto and Rosecoco. The normalized expression values of GolS1 (*PvGolS1*), GolS2 (*PvGolS2*), GolS3 (*PvGolS3*), RS1 (*PvRS1),* RS2 (*PvRS2*) and SS (*PvSS*) in the seed are shown during different developmental stages: 15, 20, 30 and 35 days after flowering (DAF). The expression values are log2 transformed and expressed in transcripts per million (TPM). Error bars represent the standard error.

Only a limited amount of information is available on the seed development in common bean. The RNA-seq study SRP030614 only contains one datapoint of the expression in the seeds (79 DAS, R9). To get a better understanding of the expression of the galactinol- and RFO biosynthesis genes during the seed development, an expression atlas was made by RNA-seq analysis for the common bean cultivars Canadian wonder, Pinto and Rosecoco. The expression levels were measured during early (15 DAF), mid (20 DAF) and late (30 and 35 DAF) maturation stages of seed development using four biological repeats. In all cultivars, *PvGolS1* is expressed at a much higher level in comparison with the other galactinol- and RFO biosynthesis genes for all developmental stages (Figure 5). During the early stage of the seed development (15 DAF), *PvGolS1* is expressed in all three cultivars with expression levels of 8.5 TPM (SE = 1.6) in Canadian wonder, 132.6 TPM (SE = 54.5) in Pinto and 11.1 TPM (SE = 2.7) in Rosecoco. From here, the expression level increases over time, with expression levels during the mid-maturation stage of the seed (20 DAF) of 58.3 TPM (SE = 30.1) in Canadian wonder, 445.6 TPM (SE = 77.9) in Pinto and 128.1 TPM (SE = 31.7) in Rosecoco. The highest expression of *PvGolS1* was measured in the late stage of seed development (30 and 35 DAF). Here the expression level increased up to 4684.2 TPM (SE = 212.0) in Canadian wonder and 3351.0 TPM (SE = 1107.2) in Rosecoco at 35 DAF. In Pinto, the highest expression was measured at 30 DAF, with an expression level of 1305.9 TPM (SE = 190.4). In all cultivars, expression of the other galactinol- and RFO biosynthesis genes was low to undetectable in the early and mid-maturation stages. Only a slight expression of *PvSS*, and to a minor extent *PvRS2* and *PvGolS2*, was measured during the mid-maturation stage of seed development in Pinto with expression levels of respectively, 16.8 TPM (SE = 2.1), 4.6 TPM (SE = 1.2) and 3.2 TPM (SE = 0.7). The expression of *PvGolS2* increased over time during the late stage of the seed development with the highest expression level measured at 35 DAF in Canadian wonder (154.6 TPM; SE = 11.3), Pinto (188.7 TPM; SE = 9.4) and Rosecoco (156.9 TPM; SE = 21.3). *PvRS2* was also mainly expressed during the late stage of the seed development with the highest expression level measured at 35 DAF in Canadian wonder (89.4 TPM; SE = 7.0) and Rosecoco (61.7 TPM; SE = 13.7). In Pinto, the highest expression was measured at 30 DAF (29.9 TPM; SE = 8.0). *PvGolS3* and *PvRS1* were not expressed in the seeds.

#### 2.2.3. Validation of RNA-Sequencing Results

**Figure 6:**
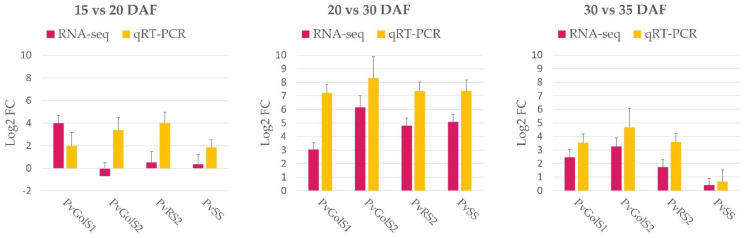
Validation of the RNA-seq data using qRT-PCR. The gene expression ratios of GolS1 (*PvGolS1*), GolS2 (*PvGolS2*), RS2 (*PvRS2*) and SS (*PvSS*) are represented as a result of the relative comparison of the different developmental stages of the seeds of *P. vulgaris* cv. Rosecoco: 15 vs. 20 days after flowering (DAF), 20 vs. 30 DAF and 30 vs. 35 DAF. These ratios are expressed as log2fold changes (Log2 FC). The housekeeping gene *β-tubulin* was used for the normalization of the qRT-PCR data [56]. Error bars represent the standard error.

The RNA-seq results of the galactinol- and RFO biosynthesis genes during the seed development were validated by measuring the expression values of the six galactinol and RFO synthase genes in the different developmental stages of the seeds of *P. vulgaris* cv. Rosecoco using qRT-PCR. When the differential gene expression levels of these genes were compared for the different developmental stages of the seed, a similar expression pattern was observed in both the RNA-seq and qRT-PCR data (Figure 6). Because most of these genes exhibit very low expression levels during the early and mid-maturation stages of seed development (15 DAF vs. 20 DAF), it is more difficult to compare the differential gene expression patterns for these stages. However, the overall correlation between the RNA-seq and qPCR results was high, indicated by a Spearman’s rank correlation coefficient (r_s_) of 0.77. Especially when comparing the differential gene expressions during the mid and late maturity stages of the seeds, a high correlation could be seen (r_s_ = 0.93). In addition, the qRT-PCR results confirmed the absence of the expression of *PvGolS3* and *PvRS1* in the seeds during the seed development. 

## 3. Discussion

A lot of progress has already been made for *G. max* in the identification of the genes involved in the RFO biosynthesis pathway. Our study confirms the results of the earlier identified genes involved in the RFO pathway. Le et al. (2020) identified the same six GolS genes and showed that *GmGolS1_A* and *GmGolS2_B* both contribute to the production of RFOs through two CRISPR/Cas9-mediated knockout lines [45]. Dierking and Bilyeu (2008) identified the same three RS genes and Valentine et al. (2017) proved that *GmRS2* contributes to the production of raffinose with the use of a soybean line in which *GmRS2* was silenced [7,14]. Qiu et al. (2015) identified and characterized the same SS gene as in our study [57]. This validates the performance of our bioinformatics pipeline and no new additional galactinol and RFO biosynthesis genes were found in *G. max*. In contrast, the galactinol- and RFO synthase genes have not yet been identified in *P. vulgaris* until now. We identified three GolS genes in *P. vulgaris*, named *PvGolS1*, *PvGolS2* and *PvGolS3. PvGolS1* consists of 3 exons, while both *PvGolS2* and *PvGolS3* contain 4 exons. This is in accordance with the genetic structures of other well-characterized GolS genes [23,51,57,58,59,60]. Previous research showed that AtSIP2 in *A. thaliana* was incorrectly annotated as an RS gene and encodes an alkaline α-galactosidase with substrate specificity for raffinose [61]. We found that in *P. vulgaris*, many genes annotated as potential RS genes in the databases were incorrectly annotated and had similar gene sequences and structures as those of alkaline α-galactosidases. We identified two RS genes, *PvRS1* and *PvRS2*, and one SS gene, *PvSS*, in *P. vulgaris*. Their genetic structure corresponds with well-characterized RS and SS genes of species other than *P. vulgaris* [40,51,52,57,62]. The presence of *PvSS* and *PvGolS1* in *P. vulgaris* was also reported by Moghaddam et al. (2018) in a Genome-Wide Association Study (GWAS), where they showed their involvement in the RFO biosynthesis pathway [9]. 

*G. max* and *P. vulgaris* are two closely related species within the Fabaceae family. Around 11 million years ago, *G. max* underwent a whole-genome duplication (WGD) and consistently we found, for almost all galactinol- and RFO synthase genes in *P. vulgaris*, a duplicate in *G. max* [63,64]. However, the RS1 and SS genes appear to have only one copy in *G. max* as in *P. vulgaris*. After the WGD, chromosomes were subject to rearrangements and deletions resulting in gene loss [65]. It is possible that duplicates of the RS1 and SS genes were lost from the *G. max* genome during these rearrangements. We subdivided the different galactinol and RFO biosynthesis genes into different classes based on the sequence and structure similarity of the genes between *P. vulgaris* and *G. max*. The chromosomal location of the genes in the different classes corresponds with syntenic relationship between these two species (Figure S1 of the Supplementary Materials) [63,64]. Indeed, duplication and speciation events can lead to the formation of genes with novel functionalities or different expression patterns. However, the high homology between the newly identified genes indicates a high probability that these genes still have the same function which is supported by the research done in *G. max* [14,45,57]. When the expression patterns are compared in *G. max* cv. Wm82 it is interesting to see that in general the different classes of galactinol- and RFO biosynthesis genes cluster together in the expression heatmap generated from the re-analysis data of the RNA-seq study. This indicates that the galactinol- and RFO biosynthesis genes within one class have a conserved expression pattern. This is further supported when the expression patterns of the different classes were compared between *G. max* and *P. vulgaris*. However, not all plant tissues are equally represented in the RNA-seq re-analysis studies of *P. vulgaris* cv. BAT93 and *G. max* cv. Wm82, which needs to be taken into account. Our novel RNA-seq study measured the expression levels of the galactinol- and RFO synthase genes in the seeds of *P. vulgaris* cv. Canadian wonder, cv. Rosecoco and cv. Pinto during the early, mid and late maturation stage of the seed. A similar expression pattern can be seen for all the galactinol- and RFO biosynthesis genes in these three cultivars and hence the expression patterns were only validated in one variety of *P. vulgaris*, namely, cv. Rosecoco.

In multiple plant species such as *Coffea arabica* and *Zea mays*, tissue-specific expression patterns for GolS genes have been observed [58,66,67]. In *C. arabica*, for example, higher expression levels of *CaGolS1* were measured in the leaves in comparison with *CaGolS2* and *CaGolS3*. Little to no expression was measured for *CaGolS2* in all tissues and *CaGolS3* was mainly expressed in the roots and flowers [66]. In this paper, we show this is also the case for *G. max* and *P. vulgaris*. In *G. max*, *GmGolS1_A* and *GmGolS1_B* are both highly expressed during the mid-maturation stage of the seeds, with *GmGolS1_A* being expressed two times higher than *GmGolS1_B*. At this stage, both genes are expressed more in soybean in comparison with the other galactinol- and RFO synthase genes indicating that they have a possible significant role during seed development. Indeed, their involvement in the RFO biosynthesis pathway during seed development in soybean is confirmed by Le et al. (2020) [45]. Knocking out *GmGolS1_A* in *G. max* resulted in a significant reduction of the total RFO content in the seed, whereas the double knockout line of both *GmGolS1_A* and *GmGolS1_B* further reduced the total RFO content but to a lesser extent. This indicates that especially *GmGolS1_A* is important during seed development. The function of the GolS1 genes is not limited to seed development. Both GolS1 genes in *G. max* show expression in other organs, including the roots, hypocotyl, stem node, leaves and flower during the different developmental stages, however, *GmGolS1_B* shows a higher expression level in these tissues in comparison with *GmGolS1_A*. In *P. vulgaris*, *PvGolS1* also shows a relatively high expression level during seed development, especially in the mid and late stages of seed maturation. This was observed in our novel RNA-seq study in *P. vulgaris* cvs. Rosecoco, Pinto and Canadian wonder and was validated with qRT-PCR in *P. vulgaris* cv. Rosecoco. The transcript levels of *PvGolS1* were higher than other galactinol- and RFO synthase genes. This result is uniform between the RNA-seq re-analysis data of *P. vulgaris* cv. BAT93 and our RNA-seq data. Besides the expression in the seeds, *PvGolS1* also shows expression in all vegetative tissues of *P. vulgaris* during the different developmental stages. The expression of *PvGolS1* is especially high in the hypocotyl, epicotyl, stem, leaves and roots which corresponds with the expression pattern of the GolS1 genes in *G. max*. This indicates that the general expression pattern of the GolS1 class genes is conserved between *G. max* and *P. vulgaris.* However, in *G. max*, *GmGolS1_A* is mostly active during the seed development, whereas *GmGolS1_B* is mostly active in the other vegetative tissues. The GolS2 genes in *G. max* are the only genes that do not cluster together in the expression heatmap of *G. max* cv. Wm82 and show a distinct expression pattern compared to each other. *GmGolS2_A* is mainly expressed in the flower, whereas *GmGolS2_B* shows its highest expression in the seeds during the seed mid-maturation stage. Both genes are to a lesser extent expressed in the hypocotyls. In *P. vulgaris*, *PvGolS2* shows a high expression in the primary root during the emergence stage and to a lower extent in the hypocotyl, epicotyl and stem. During seed development, *PvGolS2* is expressed mainly in the late stage of seed development in all three *P. vulgaris* cultivars. These results suggest that *PvGolS2* of *P. vulgaris* and *GmGolS2_B* of *G. max* play a role during the seed development, whereas *GmGolS2_A* seems to have a specific role in the flower in *G. max*. It is interesting to note that the GolS3 genes in *P. vulgaris* and *G. max* do not play a major role during the seed development in comparison with the other GolS genes. In *G. max*, only a low expression can be seen in the seeds whereas in *P. vulgaris* no expression of *PvGolS3* was detected in the RNA-seq re-analysis data of cv. BAT93 and the novel RNA-seq analysis data of cvs. Rosecoco, Pinto and Canadian wonder. The GolS3 genes are primarily expressed in the roots, cotyledon and hypocotyl in both *G. max* and *P. vulgaris*. In *G. max*, *GmGolS3_A* and *GmGolS3_B* are also expressed in the flower and leaves. The GolS3 genes show, in comparison with the other galactinol and RFO synthase genes, the highest expression in the roots, indicating a possible function for this class in the roots. 

**Figure 7:**
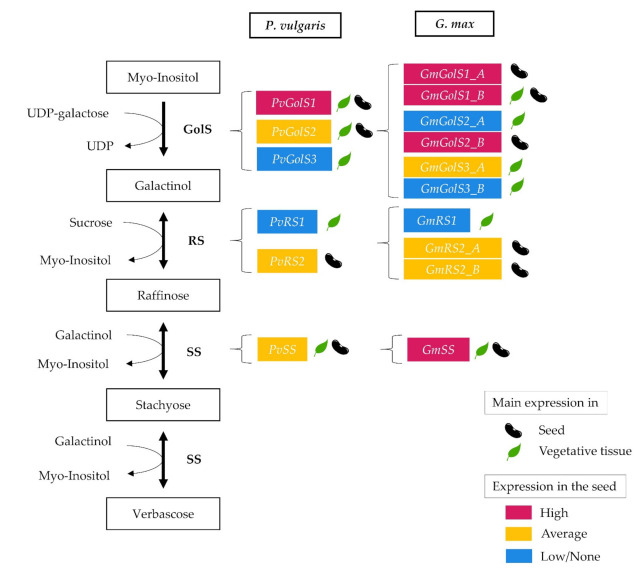
Overview of the RFO metabolic pathway and corresponding genes in *P. vulgaris* and *G. max.* The galactinol- and RFO synthase genes for *P. vulgaris* and *G. max* are shown to the right of the metabolic pathway. The main expression of these genes, either in the seed or the vegetative tissue, is indicated behind the gene names and the expression level of these genes in the seed are indicated in color, ranging from red (high), orange (average) to blue (low or no expression).

Tissue-specific expression patterns can also be seen for the RS genes. The RS1 gene in *G. max* and *P. vulgaris* is mainly expressed in the leaves and hypocotyl. In *P. vulgaris*, *PvRS1* is additionally expressed in the primary root during the emergence stage. Expression of the RS1 gene cannot be seen in either species during seed development. In contrast, the RS2 genes are mainly active in the seeds during the mid and late maturation stage in *P. vulgaris* and *G. max*. In soybean and chickpea seeds, raffinose mainly accumulates in the late stage of seed development which corresponds with the expression pattern seen for the RS2 genes in common bean and soybean [34,68,69]. Furthermore, research done by Valentine et al. (2017) showed that by silencing the *GmRS2_A* gene in *G. max*, the raffinose and stachyose content in the seeds significantly decreased [14]. In *P. vulgaris*, expression of *PvRS2* can also be seen during the emergence stage of the plant, mainly in the primary leaves. However, no expression of *PvRS2* can be seen in the later developmental stages. This suggests that the RS1 genes in *P. vulgaris* and *G. max* are mainly active in the vegetative tissue, whereas the RS2 genes are primarily active in the seeds. These results are partly in contrast with the qRT-PCR results of Dierking and Bilyeu (2008) that showed comparable expression levels for *GmRS1* and *GmRS2_A* genes in the seeds and leaves of *G. max* [7]. These authors further showed that *GmRS1* was not associated with the seed raffinose and stachyose content, which corresponds with our observations. They suggested that that *GmRS2_B* is probably not associated with the seeds’ raffinose and stachyose content because *GmRS1* and *GmRS2_B* are located on the same chromosome. However, our observations suggest that *GmRS2_B* is also responsible for the production of raffinose in the seeds. The SS single-copy gene in *G. max* and *P. vulgaris* mainly shows expression in the primary and trifoliate leaves, the hypocotyl and cotyledon after emergence. It also shows expression in the seeds, primarily during the seeds’ mid and late maturation stage. In *P. vulgaris*, *PvSS* is also expressed in the primary root during the emergence stage. The elevated expression level of *SS* in the seeds during the mid and late maturation stage of the seed development in *G. max* and *P. vulgaris* corresponds with the observations made in the seeds of soybean and chickpea where the stachyose content also increased during this period [34,68,69]. The combined results of the gene expression atlases demonstrate that the different classes of galactinol- and RFO synthase genes show tissue-specific expression patterns in soybean and common bean grown under standard conditions (Figure 7). The different classes of galactinol- and RFO synthase genes most likely also show a specific expression pattern during abiotic stress conditions [23,26,31,32]. For example, in *C. arabica*, the GolS gene *CaGolS1* showed an increased expression level during drought, heat and salt stress. The GolS gene *CaGolS1*, which was almost not expressed during standard growth conditions, had elevated transcript levels during drought and salt stress and *CaGolS3* was primarily expressed during drought stress [66]. Further research in *P. vulgaris* and *G. max* could give a better understanding of the role of the different galactinol- and RFO synthase classes during abiotic stress. 

Lowering the amount of RFOs in the seeds could improve the nutritional quality of these beans and would solve the discomforts associated with their consumption [14]. However, RFOs also play an important role in the seed, protecting it against desiccation, providing longevity during storage and as an energy source during germination [31,32,33,34,35,36,37,38]. In *A. thaliana*, it was shown that seed vigor is not correlated to the absolute amount of a specific RFO molecule but rather to the total amount of RFOs and the ratio of RFOs to sucrose [51]. Contrastingly, when a wild-type soybean was compared with a low RFO variety, no significant difference was observed in terms of germination rate [39]. When choosing target genes this should be taken into account and further research should determine the optimal balance between seed health and the benefits for the consumer. Furthermore, RFOs are also needed for sugar transport through the phloem and protect the plant against abiotic stress. Gene targets that would not compromise normal plant development and functioning should be selected. One possible strategy is the targeting of seed-specific galactinol- and RFO synthase genes responsible for the production of RFOs in the seeds. With the current insights of the expression patterns, suitable candidate genes to alter the expression levels of the galactinol- and RFO synthase genes are proposed to lower the amount of RFOs in the seeds. In soybean, the *GolS* gene, *GmGolS1_A*, and the *RS* genes, *GmRS2_A* and *GmRS2_B*, form interesting genes to target because of their expression patterns, indicating that their main activity is during seed development (Figure 7). In common bean, *PvRS2* is the only gene with a seed-specific expression pattern, making it an interesting candidate to knock out. The GolS1 gene, *PvGolS1*, shows a very high expression level in the seeds of the common bean, indicating its importance in the accumulation of galactinol in the seeds. However, this gene is also expressed in many other plant tissues. Targeting this gene could potentially lead to unwanted phenotypic side effects. However, this does not necessarily have to be the case as was observed in a soybean double mutant where both the GolS1 genes were knocked out, with no adverse effects on the plants’ growth [45]. Besides the formation of stachyose, SS also facilitates the formation of galactinol cyclitols which additionally contribute to the digestive problems associated with RFOs [37,42]. Stachyose is also the main RFO compound present in common bean and soybean seeds. This makes the SS gene also an interesting candidate gene [1,69]. Targeting a combination of these proposed GolS, RS and SS genes will most likely lead to the best result to lower the quantities of RFOs in the seeds. This should increase the nutritional value and decrease the flatulence and stomach discomforts associated with the consumption of common bean and soybean.

## 4. Materials and Methods

### 4.1. Plant Material

Seeds from *P. vulgaris* cvs. Pinto, Rosecoco and Canadian wonder, obtained from the Kenya Agricultural and Livestock Research Organization (Nairobi, Kenya) were grown in a greenhouse (Brussels, Belgium) under a 16/8 h light/dark regime. Seeds were harvested at 15, 20, 30 and 35 days after flowering using the flash freezing method with liquid nitrogen and stored at −80 °C.

### 4.2. In Silico Identification of the Galactinol and RFO Biosynthetic Genes in Phaseolus vulgaris and Glycine max

The amino acid sequences of well-characterized galactinol- and RFO synthase enzymes of *Arabidopsis thaliana* (AT2G47180.1, AT1G56600.1, AT1G09350.1, AT1G60470.1, AT5G23790.1, AT4G26250.1, AT1G60450.1, AT5G30500.1, NP_198855.1, NP_192106.3), *Cicer arietinum* (AMP59727.1, AMP59729.1), *Pisum sativum* (CAD20127.2), *Oryza sativa* (XP_015621501.1), *Zea mays* (NP_001354805.1) and *Vigna angularis* (CAB64363.1) were initially used as queries against the protein and gene databases of the National Center for Biotechnology Information (NCBI) and Phytozome databases to search for the orthologs in *P. vulgaris* and *G. max* using BLASTP and TBLASTN (Table S1) [23,40,51,52,60,61,62,70,71,72,73,74,75,76,77,78]. The resulting gene/protein sequences were further used to perform a combination of BLASTP, TBLASTN and BLASTN to find all potential galactinol and RFO biosynthetic genes in *P. vulgaris* and *G. max*. A multiple sequence alignment of the amino acid sequences was made using the MUSCLE algorithm in MEGA X (v10.2.4) and screened for conserved motifs to distinguish the different RFO synthase enzymes [48]. Two conserved motifs (FMxLGTEAxxLG and SGDPxGTxWLQGCHMVHC) were used by the motif search method of MEGA X(v10.2.4) to distinguish RS from alkaline α-galactosidase [51]. To further distinguish SS from RS the presence of a specific amino acid insert, only present in SS as described in Peterbauer et al. (1999), has been evaluated through multiple sequence alignment [52]. Sequence similarity and identity were evaluated and compared with other well-characterized galactinol- and RFO synthase enzymes of *A. thaliana* (AT2G47180.1, AT1G56600.1, AT1G09350.1, AT1G60470.1, AT5G23790.1, AT4G26250.1, AT1G60450.1, AT5G30500.1, NP_198855.1, NP_192106.3), *C. arietinum* (AMP59727.1, AMP59729.1), *P. sativum* (CAD20127.2), *O. sativa* (XP_015621501.1), *Z. mays* (NP_001354805.1) and *V. angularis* (CAB64363.1). Isoforms where predicted based on the annotation of the Phytozome database. To evaluate the evolutionary relationship, a phylogenetic tree was made in MEGA X (v10.2.4) using the amino acid sequences of the different galactinol and RFO biosynthetic and hydrolytic enzymes of *P. vulgaris*, *G. max* and well-annotated species *A. thaliana* (AT2G47180.1, AT1G56600.1, AT1G09350.1, AT1G60470.1, AT5G23790.1, AT4G26250.1, AT1G60450.1, AT5G30500.1, NP_198855.1, NP_192106.3, OAP05273.1, NP_191190.2, NP_001031855.1), *C. arietinum* (AMP59727.1, AMP59729.1), *P. sativum* (CAD20127.2), *O. sativa* (XP_015621501.1), *Z. mays* (NP_001354805.1, AAQ07251.2, NP_001105794.2, NP_001105775.2) and *V. angularis* (CAB64363.1), *Cucumis melo* (AAM75139.1, AAM75140.1) and *Solanum lycopersicum* (AAF04591.1) using the neighbor-joining algorithm combined with a bootstrap test of 1000 replicates (Table S1) [46,48,51,58,79]. The Poisson correction method was used to compute the evolutionary distances with the number of amino acid substitutions per site as a unit [49]. The phylogenetic tree was drawn to scale using the calculated evolutionary distances as branch lengths. The features of the galactinol and RFO biosynthesis genes were visualized using Gene Structure Display Server 2.0 (GSDS) software [50]. 

### 4.3. Transcriptome Analysis of the RFO Biosynthesis Pathway

#### 4.3.1. Expression Atlas of Galactinol- and RFO Biosynthesis Genes in *G. max* and *P. vulgaris* by RNA-seq Re-Analysis

The sequence read archive (SRA) of the International Nucleotide Sequence Database Collaboration (INSDC) was screened for publicly available RNA-seq studies that could be used for the creation of an expression atlas of *G. max* and *P. vulgaris* under normal conditions. For *P. vulgaris*, study SRP030614 containing RNA-seq data of genotype BAT93, as described in Vlasova et al. (2016), was used for the creation of an expression atlas [53]. The common bean plants of this study were grown under a 16 h light and 8 h dark photoperiod at ±25 °C and 80% humidity. The study consists of 61 run accession files (SRR) containing raw read files (FASTQ) comprising 453 247 Mbases. The RNA-seq data of study SRP038111, as described in Shen et al. (2014), was used for the creation of an expression atlas for *G. max* cv. Wm82 [54]. The soybean plants in this study were grown during the growing season at the experimental station of the Institute of Genetics and Developmental Biology of the Chinese Academy of Sciences (38°06′56″ N 114°32′00″ E). This study consists of 28 SRR files containing raw read files (FASTQ) comprising 181 065 Mbases. FASTQC (v0.11.8) was used for the quality control of the FASTQ files. The raw read files were converted into gene count files using a customized Bash script adapted from Kiekens et al. (unpublished; manuscript in preparation) [80]. In short, raw read files were trimmed using Sickle (v1.33) with the threshold value for the fragment length set at 35 bp and the threshold for the quality score set at 30 [81]. STAR (v2.6.0) was used to map the trimmed reads on the Wm82.a2.v1 genome assembly for *G. max* and the Pvulgaris_442_v2.1 genome assembly for *P. vulgaris* (http://phytozome.jgi.doe.gov/; accessed on 15 May 2020) [74,82]. The number of sequence reads per gene was calculated using HTSeq (v0.9.1) [83]. The resulting count files were normalized for both the gene length and sequencing depth and were expressed in transcripts per million (TPM) [84]. Heatmaps were created using Pheatmap (v1.0.12) within R studio (v1.4.1106) [85]. 

#### 4.3.2. Expression Atlas of Galactinol- and RFO Biosynthesis Genes during the Seed Development in *P. vulgaris* by *De Novo* RNA-seq Analysis

At four different developmental stages (15, 20, 30 and 35 days after flowering (DAF)) seed samples of *P. vulgaris* cvs. Pinto, Rosecoco and Canadian wonder were used to extract RNA from using the RNeasy PowerPlant Kit (Qiagen, Hilden, Germany, CAT #13500-50). The quality and quantity of the RNA samples were measured using a Thermo Scientific (Waltham, MA, USA) NanoDrop 1000 Spectrophotometer and the RNA integrity was determined using the bleach gel electrophoresis protocol described by Aranda et al. [86]. Four biological repeats for each developmental stage were sent for sequencing to the Genomics Core facility (Leuven, Belgium). The library was prepared using the QuantSeq 3′ mRNA-Seq Library Prep Kit (FWD) (Lexogen, Wien, Austria, CAT #015.96) and Illumina’s HiSeq4000 was used for sequencing. The resulting raw read files were converted into gene count files using a customized Bash script based on the optimized QuantSeq FWD/REV Data Analysis Pipeline (Available at https://www.lexogen.com/wp-content/uploads/2021/01/015UG108V0310_QuantSeq-Data-Analysis-Pipeline-on-BlueBee-Platform_2021-01-20.pdf; accessed on 15 Aug 2020). The quality of the raw read files was checked using FASTQC (v0.11.8) and trimmed using BBDuk from the BBmap suite (v38.50b, settings: -k13 -ktrim r -useshortkmers t -mink 5 -qtrim r -trimq 10 -minlength 20). FastQC (v0.11.8) was also used to check the quality of the trimmed files. Mapping of the reads on the Pvulgaris_442_v2.1 genome assembly (*P. vulgaris* v2.1, DOE-JGI and USDA-NIFA, http://phytozome.jgi.doe.gov/; accessed on 15 May 2020) was performed using STAR (v2.6.0) after which Qualimap 2 (v2.2.1) was used to control the quality of the mapping [74,82,87]. The number of sequence reads per gene was calculated using HTSeq (v0.9.1) [83]. The resulting count files were normalized for both the gene length and sequencing depth and were expressed in transcripts per million (TPM) [84]. A more detailed analysis of the RNA-seq data will be published elsewhere by Toili et al. (unpublished; manuscript in preparation) [88].

### 4.4. Validation of RNA-Sequencing Results in P. vulgaris cv. Rosecoco

The results of the novel RNA-seq study were validated in one variety of *P. vulgaris*, namely, cv. Rosecoco. In this variety, the expression levels of the galactinol- and RFO biosynthesis genes were measured during different stages of the seed development using qRT-PCR. The primers used for qRT-PCR were designed using primer-BLAST [89]. Standard settings were used for the product size ranging from 70 to 200 bases and melting temperature ranging from 58.0 to 62.0 °C with an optimum of 60.0 °C and a maximum temperature difference of 2 °C. The concentration of dNTPs was set to 0.4 and the concentration of divalent cations was set to 3.0 in the advanced settings. To ensure no genomic DNA could be amplified, only primers that spanned an exon-exon junction were chosen. Standard curves were made to verify the specificity and amplification efficiency of each primer pair. *β-tubulin* was used as a reference gene [56]. An overview of the primers used for qRT-PCR can be found in Table S2 of the Supplementary Materials.

RNA samples to be used for the qRT-PCR were extracted from 15, 20, 30 and 35 DAF seeds of *P. vulgaris* cv. Rosecoco (see Section 4.3.2) and treated with RQ1 RNase-Free DNase (Promega, Madison, WI, USA, CAT #M6101) to remove genomic DNA. cDNA was synthesized using the RevertAid H Minus First Strand cDNA Synthesis Kit (Thermo Scientific, CAT #K1631). GoTaq qPCR Master Mix (Promega, CAT # A6001) was used to load the samples on the CFX96 TouchTM Real-Time PCR Detection System (Bio-Rad, Hercules, CA, USA) to perform qRT-PCR. The following settings were used: 95 °C for 3 min followed by 40 cycles of 15 s at 95 °C and 1 min at 60 °C each. The fluorescence was measured after each cycle. The dissociation was analyzed starting at 65.0 °C and increasing till 95.0 °C with increments of 0.5 °C every 5 s. After every increment, the fluorescence signal was measured. For each developmental stage, the gene expression was measured of three biological repeats. The comparative ΔCT method was used to normalize the cDNAs threshold cycle (Ct) values observed by qRT-PCR using β-tubulin as a reference gene [56,90,91]. The differential expression levels of the galactinol- and RFO synthase genes between the different developmental stages (15 vs. 20 DAF, 20 vs. 30 DAF and 30 vs. 35 DAF) were calculated using the 2^−ΔΔCt^ method [91].

Within R studio (v1.4.1106), DESeq2 (v1.30.1) was used to calculate the differential expression levels of the galactinol- and RFO synthase genes between the different developmental stages (15 vs. 20 DAF, 20 vs. 30 DAF and 30 vs. 35 DAF) using the RNA-seq count files of *P. vulgaris* cv. Rosecoco [92]. To compare the results of the RNA-seq and qRT-PCR data, the log2 fold changes were represented in a bar chart and the correlation between the RNA-seq and qRT-PCR results were calculated using Spearman’s rank correlation coefficient.

## Figures and Tables

**Table 1:** Overview of the enzymes involved in the RFO biosynthesis pathway and corresponding genes.

Species	Gene Name	Accession Number *	Function	Chromo-Some	Start (bp)	End (bp)	Protein Length (AA)	# Exons	# Transcripts
*P. vulgaris*	*PvGolS1*	Phvul.001G215300	Galactinol synthase	Chr1	47,166,933	47,168,674	339	3	2
*G. max*	*GmGolS1_A*	Glyma.03G222000		Chr3	42,494,623	42,497,111	339	3	1
*G. max*	*GmGolS1_B*	Glyma.19G219100		Chr19	47,148,225	47,150,373	335	3	1
*P. vulgaris*	*PvGolS2*	Phvul.001G223700		Chr1	47,870,097	47,872,698	327	4	1
*G. max*	*GmGolS2_A*	Glyma.03G229800		Chr3	43,172,457	43,175,687	331	4	1
*G. max*	*GmGolS2_B*	Glyma.19G227800		Chr19	47,911,130	47,914,214	330	4	2
*P. vulgaris*	*PvGolS3*	Phvul.007G203400		Chr7	32,610,928	32,612,577	326	4	1
*G. max*	*GmGolS3_A*	Glyma.10G145300		Chr10	38,014,453	38,016,396	328	4	3
*G. max*	*GmGolS3_B*	Glyma.20G094500		Chr20	33,759,417	33,761,555	324	4	1
*P. vulgaris*	*PvRS1*	Phvul.004G007100	Raffinose synthase	Chr4	519,197	523,594	763	5	1
*G. max*	*GmRS1*	Glyma.05G003900		Chr5	307,461	312,091	758	5	1
*P. vulgaris*	*PvRS2*	Phvul.009G175400		Chr9	26,053,801	26,057,657	777	5	1
*G. max*	*GmRS2_A*	Glyma.06G179200		Chr6	15,217,419	15,223,877	810	5	2
*G. max*	*GmRS2_B*	Glyma.05G040300		Chr5	3,593,378	3,598,821	782	5	1
*P. vulgaris*	*PvSS*	Phvul.001G214300	Stachyose synthase	Chr1	47,049,258	47,052,441	857	4	2
*G. max*	*GmSS*	Glyma.19G217700		Chr19	47,033,812	47,037,286	860	4	1

* Accession numbers were adopted from Phytozome v12.1 (*P. vulgaris* v2.1; *G. max* Wm82.a2.v1).

## Data Availability

The data generated and analyzed in this study is available in this paper and the Supplementary Materials. The raw reads have been uploaded to the European Nucleotide Archive database (study PRJEB45523).

## Supplementary Materials

The following are available online at https://www.mdpi.com/article/10.3390/plants10071465/s1, Figure S1: Visualization of the syntenic relationship between *P. vulgaris* and *G. max*; Table S1: Overview of the enzymes used for the identification of the galactinol- and RFO synthase genes in *P. vulgaris* and *G. max*; Table S2: Primers used for qRT-PCR.
