# Supplementary material for: Identification and Expression Analysis of the Genes Involved in the Raffinose Family Oligosaccharides Pathway of Phaseolus vulgaris and Glycine max
Source: Plants (Basel). 2021 Jul 16;10(7):1465. doi: 10.3390/plants10071465 (PMC8309293; doi:10.3390/plants10071465)
Supplement: Supplementary file 1 [file plants-10-01465-s001.zip › plants-1277156-supplementary.pdf]

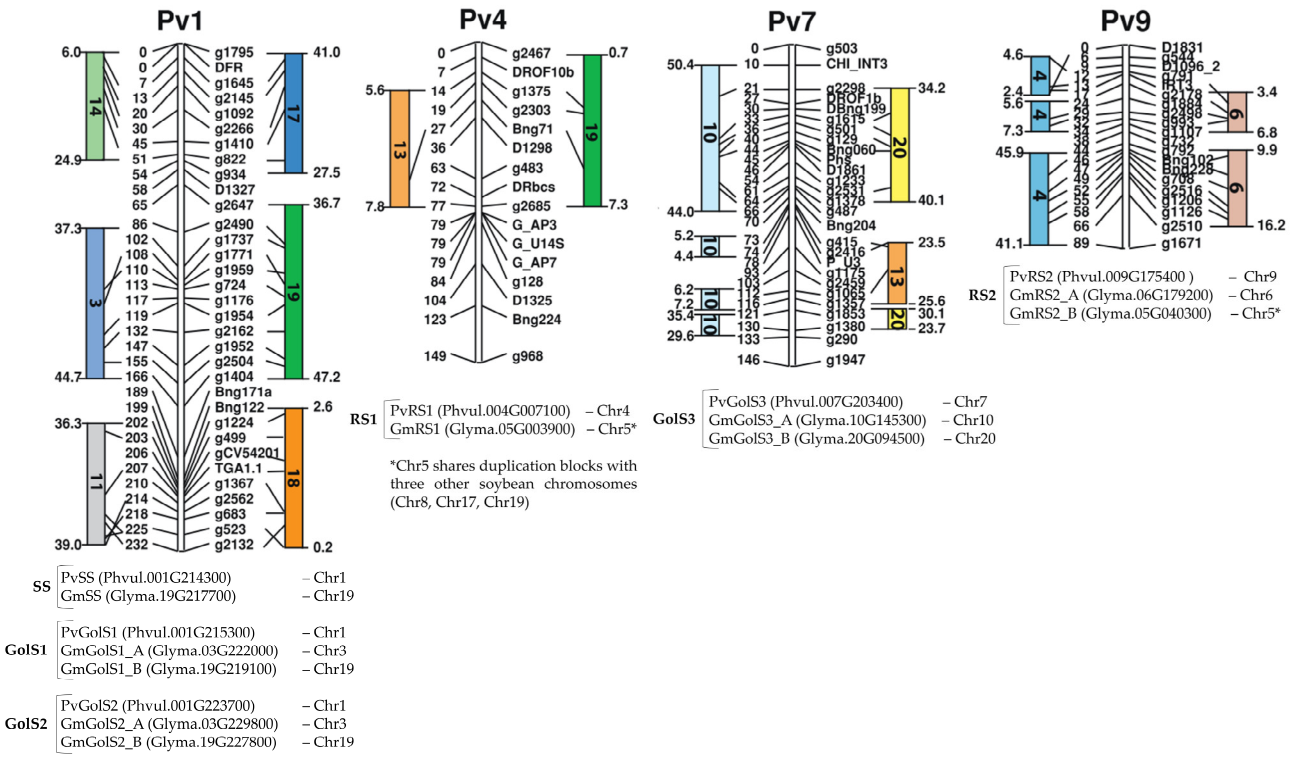

Figure S1. Visualization of the syntenic relationship between *P. vulgaris* and *G. max*. The syntenic relationship between *P. vulgaris* and *G. max* is shown for four chromosomes of *P. vulgaris* (Chr1, Chr4, Chr7 and Chr9). Each chromosomal fragment of *G. max,* linked to the *P. vulgaris* chromosome, is situated next to the *P. vulgaris* chromosome and the start and end of each fragment is shown in megabase pairs. The chromosomal location of the galactinol- and RFO synthase genes of *P. vulgaris* and *G. max* is shown underneath the genetic linkage map. The figure is adapted from McClean *et al.* (2010) [61].

Table S1. Overview of the enzymes used for the identification of the galactinol- and RFO synthase genes in *P. vulgaris* and *G. max*. The amino acid sequences of these enzymes have also been used to build the phylogenetic trees shown in Figures 1 and 2.

Species	Accession number	Name	Function	Protein lenght (AA)	Reference
Arabidopsis thaliana	AT2G47180.1	AtGolS1	Galactinol synthase	344	Taji *et al.* (2002)
	AT1G56600.1	AtGolS2		335	Taji *et al.* (2002)
	AT1G09350.1	AtGolS3		334	Taji *et al.* (2002)
	AT1G60470.1	AtGolS4		334	Taji *et al.* (2002)
	AT5G23790.1	AtGolS5		333	Taji *et al.* (2002)
	AT4G26250.1	AtGolS6		336	Taji *et al.* (2002)
	AT1G60450.1	AtGolS7		332	Taji *et al.* (2002)
	AT5G30500.1	AtGolS10		328	Sun *et al.* (2013)
Cicer arietinum	AMP59727.1	CaGolS1		339	Salvi *et al.* (2018)
	AMP59729.1	CaGolS2		325	Salvi *et al.* (2018)
Arabidopsis thaliana	NP_198855.1	AtRS	Raffinose synthase	783	Egert *et al.* (2013)
Pisum sativum	CAD20127.2	PsRS		798	Peterbauer *et al.* (2002)
Oryza sativa	XP_015621501.1	OsRS		840	Li *et al.* (2007)
Zea mays	NP_001354805.1	ZmRS		790	Li *et al.* (2017)
Arabidopsis thaliana	NP_192106.3	AtSS	Stachyose synthase	857	Gangl *et al.* (2015)
Vigna angularis	CAB64363.1	VaSS		857	Peterbauer *et al.* (1999)

Arabidopsis thaliana	OAP05273.1	AtSIP2		773	Shaun Peters *et al.* (2010)
	AAQ07251.2	ZmAGA1		756	Zhao *et al.* (2006)
Zea mays	NP_001105794.2	ZmAGA2	Alkaline α-galac-	843	Zhao *et al.* (2006)
	NP_001105775.2	ZmAGA3	tosidase	747	Zhao *et al.* (2006)
Cucumis melo	AAM75139.1	CmAGA1		754	Carmi *et al.* (2003)
	AAM75140.1	CmAGA2		772	Carmi *et al.* (2003)
Arabidopsis thaliana	NP_191190.2	AtAGAL3		437	Imaizumi *et al.* (2017)
Solanum lycopersicum	AAF04591.1	SlAGAL	α-galactosidase	409	Tsaniklidis *et al.* (2016)
Arabidopsis thaliana	NP_001031855.1	AtAGAL2		370	Imaizumi *et al.* (2017)

Table S2. Primers used for qRT-PCR.

Accession number	Function	Forward Primer	Tm (°C)	Reverse Primer	Tm (°C)	Amplicon length (nt)
Phvul.001G215300	Galactinol synthase	$^{5'}$CGCGTAGGAG ATGACTTTTG$^{3'}$	60.5	$^{5'}$GCACATGGG TGAGTAGATTG$^{3'}$	60.4	103
Phvul.001G223700	Galactinol synthase	$^{5'}$ACAGTGCCCTA ATAAAGTTCAG$^{3'}$	60.4	$^{5'}$GTTCAGAAA GTCCTGCTCAG$^{3'}$	60.2	168
Phvul.007G203400	Galactinol synthase	$^{5'}$GAATTTGGGA GTTTGTGGAG$^{3'}$	58.9	$^{5'}$AGAAGTAGTTAT CAGGCAAGTC$^{3'}$	59.6	161
Phvul.004G007100	Raffinose synthase	$^{5'}$GACAATATTT CGGTGTGCGT$^{3'}$	61	$^{5'}$TAGAAGGTTGA AGCTTCCCA$^{3'}$	60.9	155
Phvul.009G175400	Raffinose synthase	$^{5'}$GACCCATTTAC GTTAGTGATTGTG$^{3'}$	61.8	$^{5'}$CACCTGTATGTT TGTTGAGATTCC$^{3'}$	61.8	131
Phvul.001G214300	Stachyose synthase	$^{5'}$CTCATTAAGAAG CTTGTCTTCCC$^{3'}$	61.1	$^{5'}$CTCCTCCATACT TATTGAAGTTCC$^{3'}$	60.5	144
CV530631 (Pereira *et al.*, 2017)	β-Tubulin	$^{5'}$CCGTTGTGGA GCCTTACAAT$^{3'}$	62.4	$^{5'}$GCTTGGGGTC CTGAAACAA$^{3'}$	62.4	117
